# Adherence and Engagement With a Cognitive Behavioral Therapy–Based Conversational Agent (Wysa for Chronic Pain) Among Adults With Chronic Pain: Survival Analysis

**DOI:** 10.2196/37302

**Published:** 2022-05-23

**Authors:** Chaitali Sinha, Abby L Cheng, Madhura Kadaba

**Affiliations:** 1 Wysa Boston, MA United States; 2 Division of Physical Medicine and Rehabilitation Department of Orthopaedic Surgery Washington University in St. Louis St Louis, MO United States

**Keywords:** retention, engagement, Wysa, chronic pain, digital health, digital application, app, mental health, digital intervention, health intervention, symptom management, user engagement, conversational agent

## Abstract

**Background:**

Digital applications are commonly used to support mental health and well-being. However, successfully retaining and engaging users to complete digital interventions is challenging, and comorbidities such as chronic pain further reduce user engagement. Digital conversational agents (CAs) may improve user engagement by applying engagement principles that have been implemented within in-person care settings.

**Objective:**

To evaluate user retention and engagement with an artificial intelligence–led digital mental health app (Wysa for Chronic Pain) that is customized for individuals managing mental health symptoms and coexisting chronic pain.

**Methods:**

In this ancillary survival analysis of a clinical trial, participants included 51 adults who presented to a tertiary care center for chronic musculoskeletal pain, who endorsed coexisting symptoms of depression or anxiety (Patient-Reported Outcomes Measurement Information System score of ≥55 for depression or anxiety), and initiated onboarding to an 8-week subscription of Wysa for Chronic Pain. The study outcomes were user retention, defined as revisiting the app each week and on the last day of engagement, and user engagement, defined by the number of sessions the user completed.

**Results:**

Users engaged in a cumulative mean of 33.3 sessions during the 8-week study period. The survival analysis depicted a median user retention period (i.e., time to complete disengagement) of 51 days, with the usage of a morning check-in feature having a significant relationship with a longer retention period (*P*=.001).

**Conclusions:**

Our findings suggest that user retention and engagement with a CBT-based CA built for users with chronic pain is higher than standard industry metrics. These results have clear implications for addressing issues of suboptimal engagement of digital health interventions and improving access to care for chronic pain. Future work should use these findings to inform the design of evidence-based interventions for individuals with chronic pain and to enhance user retention and engagement of digital health interventions more broadly.

**Trial Registration:**

ClinicalTrials.gov NCT04640090; https://clinicaltrials.gov/ct2/show/NCT04640090

## Introduction

Digital health interventions offer an opportunity to reduce health care barriers for individuals with chronic pain by increasing accessibility and decreasing cost- and time-related barriers to care [1,2]. They offer the ability for patients to engage in care at the convenience of their own space and time, outside the resource constraints of specialized care [3]. There are several digital intervention delivery vehicles (e.g., internet-based interventions and health-related mobile apps), and most are based on a self-guided approach. Research supports the efficacy of self-guided digital psychosocial interventions such as cognitive behavior therapy (CBT) in improving impairment from chronic pain in adults [4-6]. However, the effectiveness of self-guided digital interventions still suffers from low retention and engagement rates [7,8].

Engagement refers to the extent of usage and the perceived desire to use the digital intervention over a prolonged time period [9]. Retention with a digital intervention refers to the proportion of users who remain active with the intervention during a specified time frame [10,11]. Top-performing health apps have an average 30-day retention rate of only 15% [12]. Furthermore, people with chronic illness such as chronic pain are almost twice as likely to drop out from self-guided digital interventions when compared to traditional, guided interventions [6]. Lack of engagement is associated with low adherence to interventions and high dropout rates [13,14]. Understanding and achieving high levels of retention and engagement with digital interventions for people with chronic illness, such as chronic pain, may lead to improved intervention effectiveness.

Key factors that support engagement and retention within in-person clinical care settings include patient-clinician agreement, patients’ perception of listening and empathy by clinicians, collaborative learning mechanisms for patients, and reminder systems [15]. Artificial intelligence (AI)-led conversational agents (CAs) apply these principles to deliver therapeutic content through interactive media such as conversations and stories.

The purpose of this study was to examine user retention and engagement with Wysa for Chronic Pain—a digital CA based on CBT principles—that is designed to improve general well-being among people with chronic pain.

## Methods

### Study Design and Participant Recruitment

This is an ancillary analysis of a pilot clinical trial that examined the feasibility of delivering a digital mental health intervention within the setting of an outpatient clinic visit for the management of chronic pain. As such, participant recruitment and eligibility criteria have previously been described in the pilot study [16].

In brief, participants were recruited between December 2020 and July 2021 from a US tertiary care orthopedic clinic that specializes in the management of chronic musculoskeletal pain. To be eligible, patients had to be 18 years or older and screen positively for symptoms of depression or anxiety by scoring 55 or greater on the Patient-Reported Outcomes Measurement Information System measures for depression or anxiety. Patients who were actively planning to start in-person mental health treatment and those without access to a mobile device were excluded from the study.

Participants were recruited in person by a study coordinator and were offered 8 weeks of complimentary access to the digital intervention (Wysa for Chronic Pain). After providing informed consent, they were given a unique code to access Wysa for Chronic Pain on any Android or iOS phone. Once the code was redeemed, an in-app consent screen appeared before app onboarding began. Throughout the study, no email or phone reminders were sent by the study team outside the app environment for the purposes of app retention or engagement.

### Ethical Considerations

The pilot feasibility study received approval from the Washington University institutional review board prior to participant recruitment (202005219), and the study was registered on ClinicalTrials.gov (NCT04640090).

### Intervention: Wysa for Chronic Pain

The Wysa for Chronic Pain digital health app is a novel intervention that was specifically developed for people with mental health concerns and coexisting chronic pain. It uses an AI-based digital conversational agent that acts as a supportive companion for users, and it also includes human “coaches” with master’s degrees in counseling, who support users in identifying appropriate therapeutic interventions.

**Figure 1:**
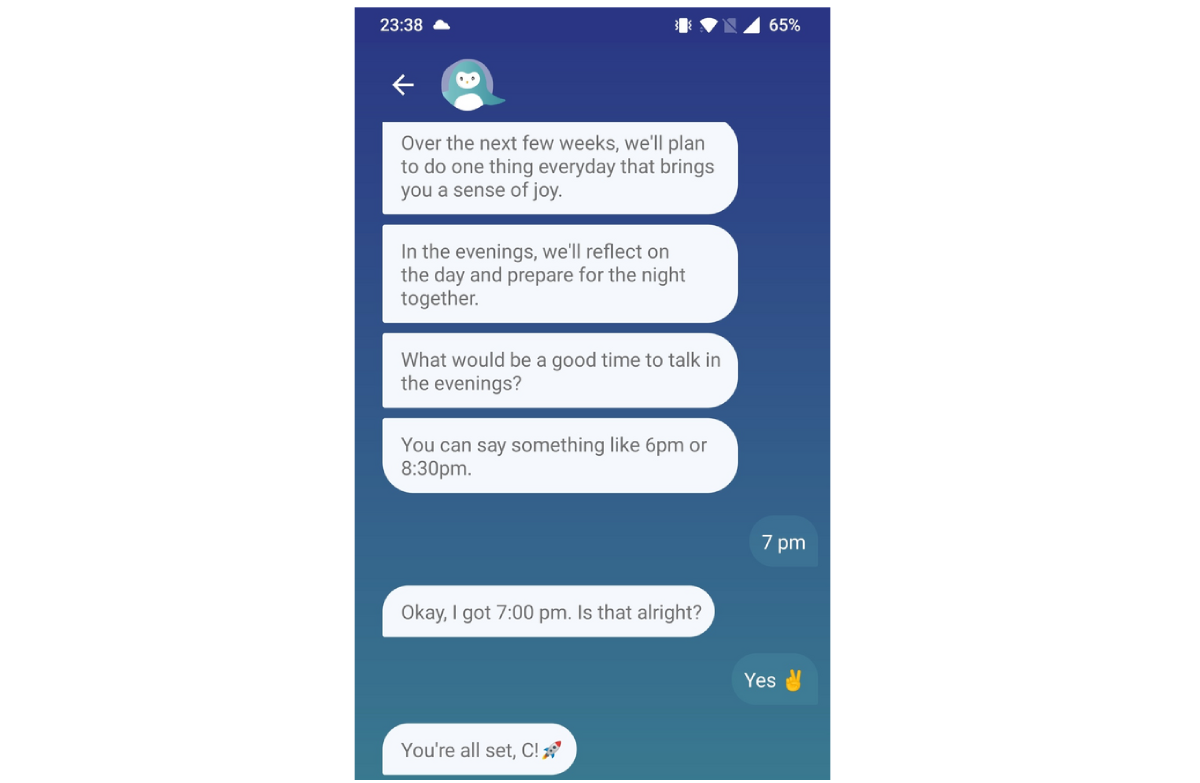
The conversational agent asks the user for a check-in time.

Wysa for Chronic Pain uses CBT principles in the app design and within digital and human conversations. A recurring and key feature of the app is morning and evening conversations (“check-ins”) with the CA (Figure 1), which occur at times chosen by the user. These conversations use behavioral activation principles.

During the morning check-ins, users report their mood on a visual analog scale and commit to engaging in an activity that day that brings them joy. Based on users’ responses, the CA suggests interventions within the app to facilitate pain acceptance and cognitive restructuring. The evening check-ins consist of mood monitoring, and a check on the completion of the planned activity. Based on users’ responses, the CA suggests additional interventions as appropriate and completes the check-in with offering users a bedtime meditation to assist with falling asleep.

The app’s engagement framework primarily rested upon notifications. The timing for the notifications would be chosen by the users, and the app would send these twice a day for the morning and evening check-ins. These would be displayed in the phone notification window, and would bring the user back to the app. Once within the app, the check-in would begin the designed structured intervention. Each week’s efforts would also be encouraged with a weekly report that offered insights and a new tool pack and further guided with a visual roadmap that would outline the path ahead.

It was hypothesized that a consistent and reliable structure that offered scheduled rewards would lead to greater app engagement and retention, along with efficacy.

### Measures

All engagement and retention data were collected automatically via the app’s usage log. To maintain user anonymity and be consistent with Wysa for Chronic Pain’s confidentiality protocols, demographic data and personally identifiable information were not collected within the app. App sessions were only counted toward engagement and retention if a user completed a conversation with the CA, conversation with the human coach, or an intervention exercise within the app. Passive events, such as opening the app or an element within the app, were not counted.

Engagement metrics included the number of sessions a user completed within (1) each week and (2) during the entire 8-week study period. Retention metrics included the following: (1) retention duration (i.e., users’ last day of engagement with the app during the 8-week study period) and (2) weekly app usage (i.e., interaction with the app at least once each week).

### Analysis

Continuous data were summarized as mean (SD) values and categorical data as n (%) values.

Previous studies have shown that quantifying user retention of an app using survival analysis is feasible. For survival analysis in this trial, the event of interest was defined as complete disengagement (i.e., the day after which a user did not return to the app within the study period). Users whose last day of engagement was after the last day of the study period were right-censored. To minimize blinding to the extent of full engagement in the study period, the Spearman and Kendall rank correlation coefficients were calculated between the total days of engagement and the last day of retention.

Kaplan-Meier nonparametric estimators for computing retention over time and the Cox proportional hazards model were used to understand the impact of morning and evening check-in notifications on user retention over the study period. All analyses included only the prespecified 8-week study period even though users could continue engaging with the app after the conclusion of the study. Data cleaning was performed using Python (version 3.6; Python Software Foundation), and all statistical analyses were performed using R (version 4.1.2; The R Foundation).

## Results

Of the 51 study participants who redeemed an app code for the trial, 49 (96%) onboarded successfully to the app.

### App Engagement

Users engaged in a mean of 4.0 (SD 0.9) sessions per week and a mean of 33.3 (SD 42) total sessions during the 8-week study period. When categorized by the type of intervention each tool delivers, the most frequently used interventions were the following: Thought Recording (19.7%), Pain Acceptance (16%), and Sleep Meditations (14.9%) (Table 1).

**Table 1:** The most frequently used interventions in Wysa for Chronic Pain.

Intervention	Usage, %
Thought Recording	19.7
Pain Acceptance	16.0
Sleep Meditation	14.9
Mindfulness	10.4
Anxiety Management	9.8
Gratitude	8.1
Motivational Stories	7.3
Calming Exercises	6.5
Social Support	2.0
Grounding	1.4

### App Retention

**Figure 2:**
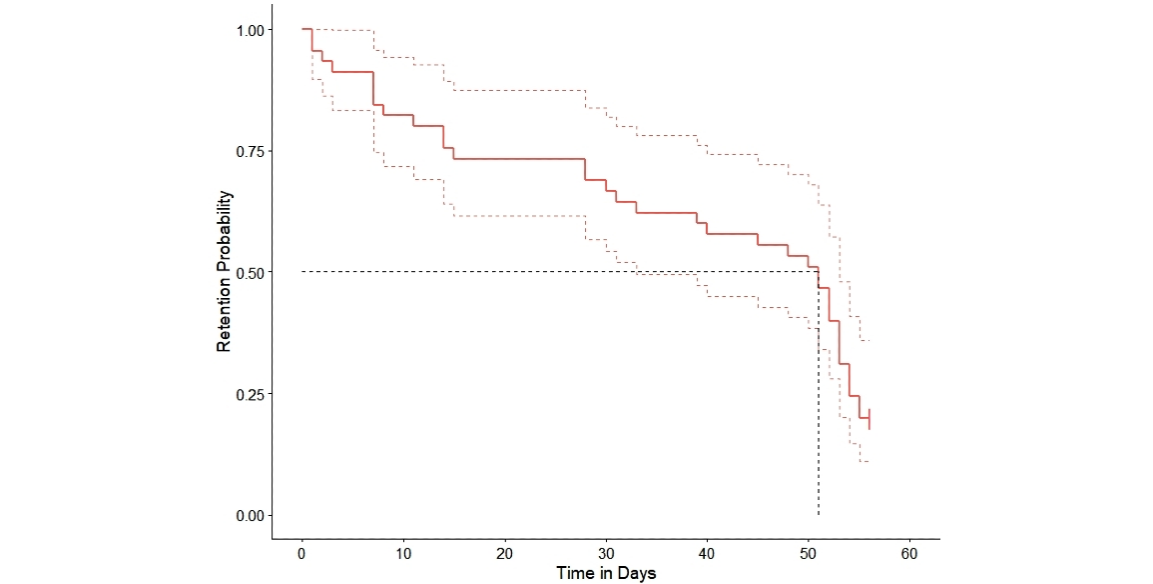
Kaplan-Meier survival curve modeling user retention in the Wysa for Chronic Pain app during the 8-week study period. Dashed lines represent 95% CIs of the survival curve. Model concordance=0.829 (SE 0.045, *P*=.001 on the Wald test).

The median user (N=49) retention period (i.e., time to complete disengagement) was 51 days (95% CI 33-53 days) (Figure 2). The retention rate at 30 days (1 month) was 70%, and 20% of users were right-censored, indicating they were still engaging with the app after completion of the prespecified 8-week study period. A strong correlation was found between users’ total number of days engaged with the app and their last day of retention (Spearman *ρ*=0.82, *P*<.001; Kendall *τ*=0.68, *P*<.001). Usage of the morning check-in was associated with a longer retention period (hazard ratio=0.89, *P*=.001) (Table 2).

**Table 2:** Cox proportional hazards model to explore the impact of engaging with morning and evening check-ins on user retention.

Variable	Hazard ratio	SE	*P* value
Morning check-in	0.894	0.035	.001^a^
Evening check-in	0.987	0.024	.59

^a^Statistically significant.

## Discussion

### Principal Findings

This study aimed to examine levels of app retention and engagement with Wysa for Chronic Pain, when used by participants managing chronic pain. The app evaluated was an AI-led mental health app (Wysa for Chronic Pain), which used proven in-person health care techniques to improve app engagement. The pilot study that used this intervention has also examined the app’s clinical efficacy while sustaining this engagement [16]. In previous studies on digital health interventions, most participants disengaged within the first week of the study, with average retention periods between 4 and 16 days [17,18]. In comparison, over 50% of users in this study continued returning to the app each week, and the median user retention period was 51 days.

These high rates of engagement and retention suggest that the participants found the app features and interventions helpful and meaningful to their everyday challenges [8]. There is a large body of research examining predictors of user engagement with digital health interventions. The literature indicates that the inclusion of behavior change techniques (e.g., self-monitoring and goal setting) [19,20] and coping strategies (e.g., mindfulness and meditation) [12] is important for the uptake and engagement with digital health interventions. Consistent with this work, our intervention included behavioral activation principles and meditation to support users. Another review based on experiential and behavioral perspectives identified content (e.g., behavior change techniques, social support, and reminders) and content delivery (e.g., professional support, personalization, and aesthetic features) as key factors that may affect engagement with digital health interventions [9]. In line with these findings, our intervention included morning and evening check-ins and personalization of content. Users received customizable notification-type reminders—a feature that has been identified as useful for engagement with digital health interventions [21,22].

Additionally, a recent study found that relational factors predict user engagement with mobile health interventions [23]. The CA in our study used machine learning and AI methods to simulate human-like behaviors and support [10,24]. Considering this, users may have developed a relational connection or a therapeutic alliance with the CA. For example, a recent study demonstrated that Wysa for Chronic Pain users’ therapeutic alliance scores were comparable to ratings from previous studies on alliance in human-delivered face-to-face psychotherapy with clinical populations [25].

Our achievement of high user retention and engagement with a CA-based, pain-customized, digital mental health intervention has clear implications for addressing issues of suboptimal engagement with digital health interventions and of improving access to care for chronic pain. Research indicates that people with chronic pain are almost twice as likely to drop out from self-guided digital interventions when compared to traditional, guided interventions [21]. Despite this, the present trial achieved high levels of retention and engagement among a sample of individuals with chronic pain. Based on the literature highlighting practical strategies and recommendations to tackle issues related to user attrition in digital health interventions, we incorporated behavior change techniques, coping strategies, and morning and evening check-ins. From our results, it is clear this combination of engagement strategies and therapeutic content has the potential to retain and engage users, which may be related to the enhanced effectiveness of the intervention. Future work should use these findings to inform the design of evidence-based interventions for individuals with chronic pain and to enhance user retention and engagement of digital health interventions more broadly.

### Limitations

The key limitations of this study are its small sample size, the lack of demographic analysis (which reduces information about the retained users), and the limited generalizability. The event of interest within the survival analysis (i.e., defined as complete disengagement) partially blinded the data to the number of breaks until the last day of engagement. With a larger sample size, a more granular analysis could evaluate each streak within the data set, leading up to the last day of engagement. Additionally, there was no comparison group, which reduced the ability to establish causality for the possible factors responsible for engagement and retention.

Given the promising results, future research should be undertaken with a larger sample size and comparative groups. It should also be determined if these results can be replicated with populations managing other health conditions and for users who were not initially introduced to the app through a medical clinic setting.

### Conclusions

This study highlights the ability to attain high retention and engagement with a mental health app that uses a digital CA and established behavioral paradigms that improve engagement with in-person therapy, specifically to deliver CBT to people with mental health concerns and coexisting chronic pain.

## Abbreviations

AI: artificial intelligence
CA: conversational agent
CBT: cognitive behavior therapy
